# Clinical data mining on network of symptom and index and correlation of tongue-pulse data in fatigue population

**DOI:** 10.1186/s12911-021-01410-3

**Published:** 2021-02-24

**Authors:** Yulin Shi, Xiaojuan Hu, Ji Cui, Longtao Cui, Jingbin Huang, Xuxiang Ma, Tao Jiang, Xinghua Yao, Fang Lan, Jun Li, Zijuan Bi, Jiacai Li, Yu Wang, Hongyuan Fu, Jue Wang, Yanting Lin, Jingxuan Bai, Xiaojing Guo, Liping Tu, Jiatuo Xu

**Affiliations:** 1 Basic Medical College, Shanghai University of Traditional Chinese Medicine, 1200 Cailun Road, Pudong, Shanghai, China; 2 Shanghai Innovation Center of TCM Health Service, Shanghai University of Traditional Chinese Medicine, 1200 Cailun Road, Pudong, Shanghai, China

**Keywords:** Fatigue, Complex network, Symptom, Index, Tongue and pulse data

## Abstract

**Background:**

Fatigue is a kind of non-specific symptom, which occurs widely in sub-health and various diseases. It is closely related to people's physical and mental health. Due to the lack of objective diagnostic criteria, it is often neglected in clinical diagnosis, especially in the early stage of disease. Many clinical practices and researches have shown that tongue and pulse conditions reflect the body's overall state. Establishing an objective evaluation method for diagnosing disease fatigue and non-disease fatigue by combining clinical symptom, index, and tongue and pulse data is of great significance for clinical treatment timely and effectively.

**Methods:**

In this study, 2632 physical examination population were divided into healthy controls, sub-health fatigue group, and disease fatigue group. Complex network technology was used to screen out core symptoms and Western medicine indexes of sub-health fatigue and disease fatigue population. Pajek software was used to construct core symptom/index network and core symptom-index combined network. Simultaneously, canonical correlation analysis was used to analyze the objective tongue and pulse data between the two groups of fatigue population and analyze the distribution of tongue and pulse data.

**Results:**

Some similarities were found in the core symptoms of sub-health fatigue and disease fatigue population, but with different node importance. The node-importance difference indicated that the diagnostic contribution rate of the same symptom to the two groups was different. The canonical correlation coefficient of tongue and pulse data in the disease fatigue group was 0.42 (*P* < 0.05), on the contrast, correlation analysis of tongue and pulse in the sub-health fatigue group showed no statistical significance.

**Conclusions:**

The complex network technology was suitable for correlation analysis of symptoms and indexes in fatigue population, and tongue and pulse data had a certain diagnostic contribution to the classification of fatigue population.

## Background

Fatigue is a state in which the body cannot initiate or maintain a certain intensity of activity, or manifest a pathological dysfunction during the initiation or maintenance of voluntary activity. It is a physiological manifestation of body's self-regulation, and a pathological result of certain diseases [1]. Fatigue is a non-specific symptom with certain heritability [2]. It is ubiquitous in sub-health and various diseases, such as Parkinson’s [3], major depressive disorder [4], schizophrenia [5], and cancer [6]. It has become one of the main factors harmful to human's physical and mental health with a serious decline in work efficiency and life quality.

Sub-health status is defined as the decline in vitality, physiological function, and adaptation capacity, but in general not meeting the diagnostic criteria for clinical or sub-clinical diseases [7, 8]. It has attracted extensive attention with the increasing incidence of sub-health in recent years. Sub-health state of fatigue is one of the most common sub-health types with that fatigue as the chief complaint. The etiology and pathogenesis of fatigue are largely unknown. There is still a lack of objective, effective, and comprehensive evaluation method for diagnosing fatigue, leading to the inability to carry out targeted interventions when fatigue occurs in the early stage of disease. Hence it is imperative to establish a comprehensive, objective evaluation method for fatigue.

In the diagnosis methods of Traditional Chinese Medicine (TCM), tongue and pulse diagnoses always play an important role in clinical diagnosis and treatment. Tongue and pulse diagnoses are comprehensive diagnostic methods based on body's overall state, suitable for comprehensive evaluation of body's functional state, and have become an important objective basis for health status evaluation and syndrome diagnosis [9]. However, traditional tongue and pulse diagnoses lack accurate classification. On the one hand, artificial intelligence and machine learning methods can quickly and accurately complete basic data cleaning and large-scale data sorting, and on the other hand, do data mining for massive tongue and pulse data. Different recognition algorithms and machine learning methods have been widely used in image recognition, target detection, natural language processing, and other fields [10–12]. Nowadays, breakthroughs have been achieved in fatigue quantification and standardization. Artificial Neural Network [13], Support Vector Machine [14], K Nearest Neighbor [15], and other machine learning methods have helped to achieve the digitalization of TCM tongue and pulse diagnoses and establish corresponding disease diagnostic models [16, 17]. The diagnostic relationship between tongue and pulse and healthy state can be better established through accurate detection, identification, and multi-dimensional quantitative analysis of tongue and pulse data to save medical resources and improve diagnosis efficiency and treatment efficacy [18–20]. Data-driven researches on fatigue diagnosis technology using tongue and pulse data have been increasing day by day. Researches based on tongue [21, 22] and pulse [23–25] of fatigue population have shown that their tongue and pulse had their own unique characteristics.

Complex network is a basic framework with high topological abstraction. Further analysis of the network through classification, screening, and other analytical methods can mine the potential rules of many clinical data. Complex network is used in the analysis of basic rules of TCM [26], network pharmacology [27–29], combined analysis of TCM syndromes and network pharmacology [30, 31], and symptom evolution [32]. Complex network is mostly used to construct qualitative network relations, which are more suitable for analyzing the symptoms involved in identifying certain syndromes and the relationship between symptoms. Fatigue is a complex concept, and it is one of the symptoms that can most reflect the interaction between psychology and physiology. As a non-specific symptom, different network relationships of fatigue symptom can be found in depression network [33], qi deficiency syndrome of coronary heart disease network [34, 35], and qi deficiency syndrome of breast cancer network [36].

Despite the progress made in current research on fatigue, there are still many problems that need to be solved. For example, studies on fatigue are relatively simple. It is essential to study the correlation of fatigue symptoms and do the combined analysis of fatigue symptoms and indexes. Besides, analysis on objective tongue and pulse data of fatigue is mostly independent. To address the above problems, in this study, 2362 physical examination population were divided into healthy controls, sub-health fatigue group, and disease fatigue group. Complex network technology was used to screen out core symptoms and Western medicine indexes. Through constructing the core symptom and index network and the core symptom-index combined network, analyzing the network structure to establish the distribution of fatigue symptoms and indexes. Simultaneously, canonical correlation analysis method was used to get the associated relationship between tongue and pulse data of disease fatigue and sub-health fatigue population. Based on symptom, Western medicine index, and tongue and pulse data, this study tried to explore the characteristics of different fatigue population from different dimensions.

## Methods

### Study design

All the 7025 people were selected in the Medical Examination Center of Shuguang Hospital affiliated to Shanghai University of Traditional Chinese Medicine from Jul. 2015 to Dec.2018. The most common diseases in fatigue population mainly include hypertension, diabetes, hyperlipidemia, and fatty liver, other diseases, such as coronary heart disease and cancer due to the lack of patients, they were not included. A total of 361 sub-health fatigue and 1529 disease fatigue population were further selected.

Four well-trained clinicians completed the diagnosis according to the diagnostic criteria of the disease. The Health Status Assessment Questionnaire (shortly, H20) and Information Record Form of Four Diagnosis of Traditional Chinese Medicine (Copyright No.: 2016Z11L025702) designed by the sub-health research group of the "863 Plan" was used to investigate fatigue syndrome. Patients with fatigue symptom were defined as disease fatigue population, those who had no obvious positive indexes in Western medicine, H20 score was between 60 and 79, and with fatigue symptom were defined as sub-health fatigue population, and those who had no obvious positive indexes in Western medicine, H20 score was between 80 and 100, and without fatigue symptom were defined as healthy controls.

**Fig.1:**
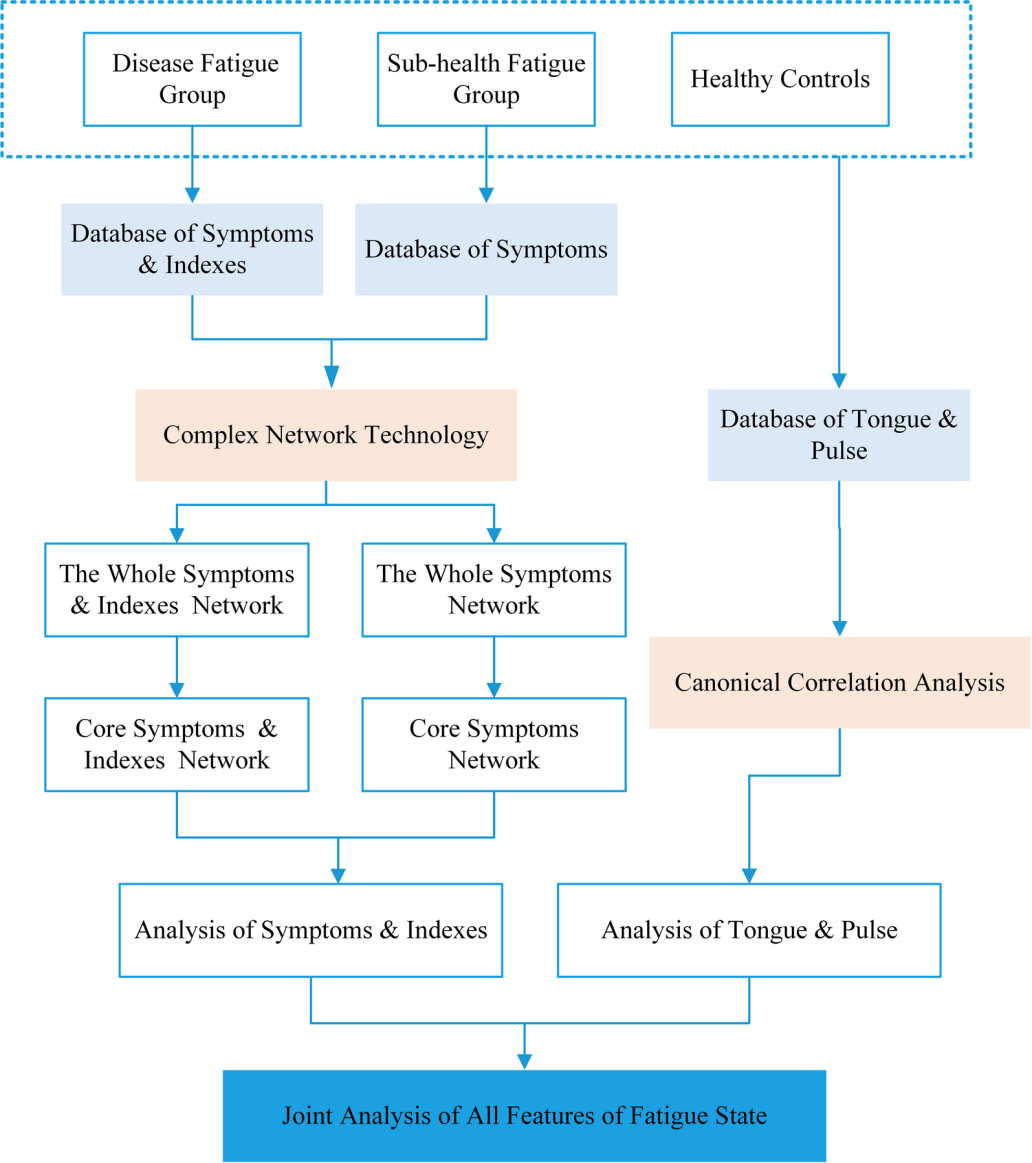
Overall flow diagram

The overall flow diagram of this study was shown in Fig. 1.

### Information on the four diagnostic methods of TCM and Physical and Chemical Indexes

**Fig.2:**
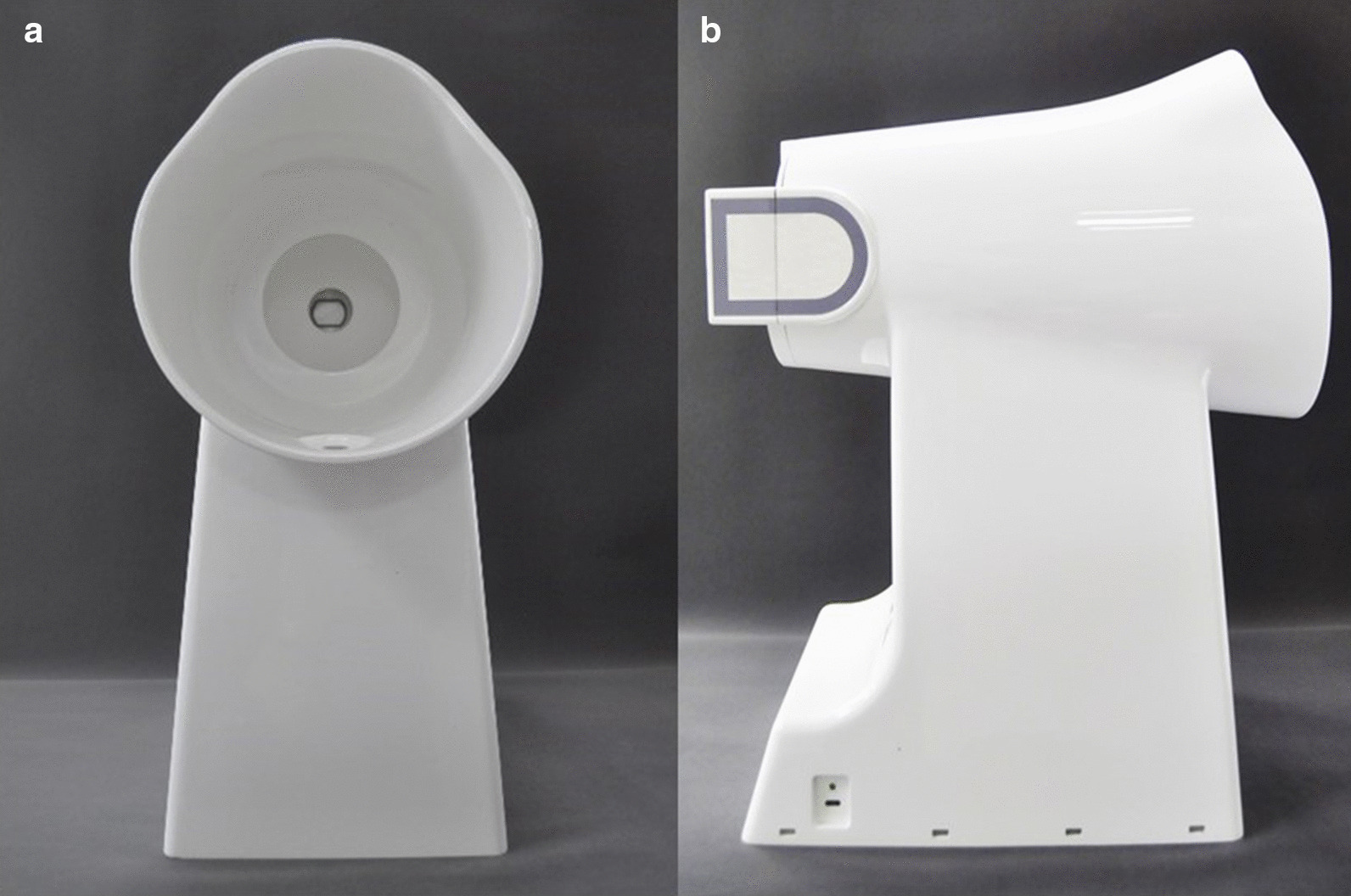
Figures of TFDA-1 tongue and face diagnosis instrument. **a** Front view, **b** Profile view

**Fig. 3:**
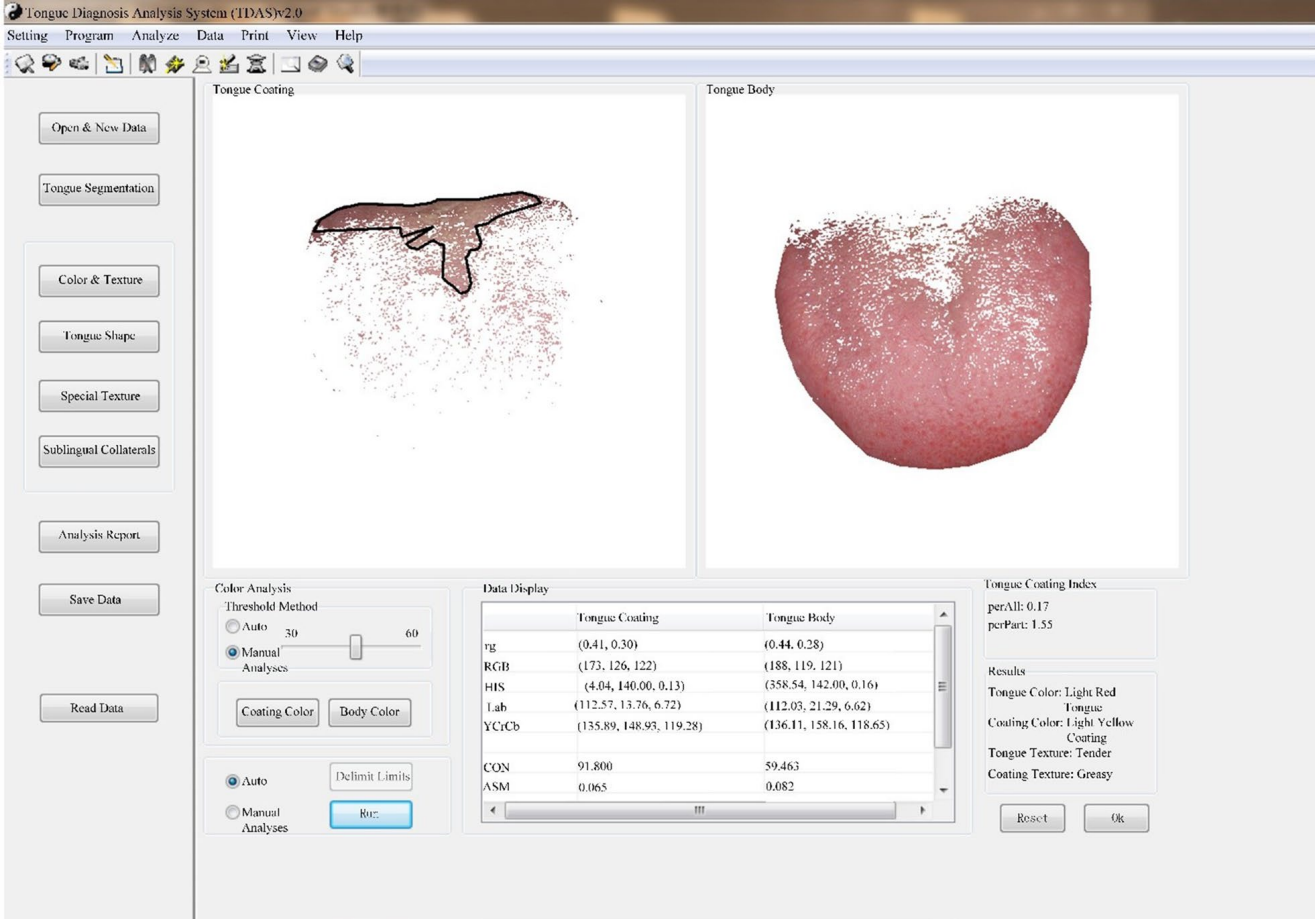
Tongue image analysis interface

**Fig. 4:**
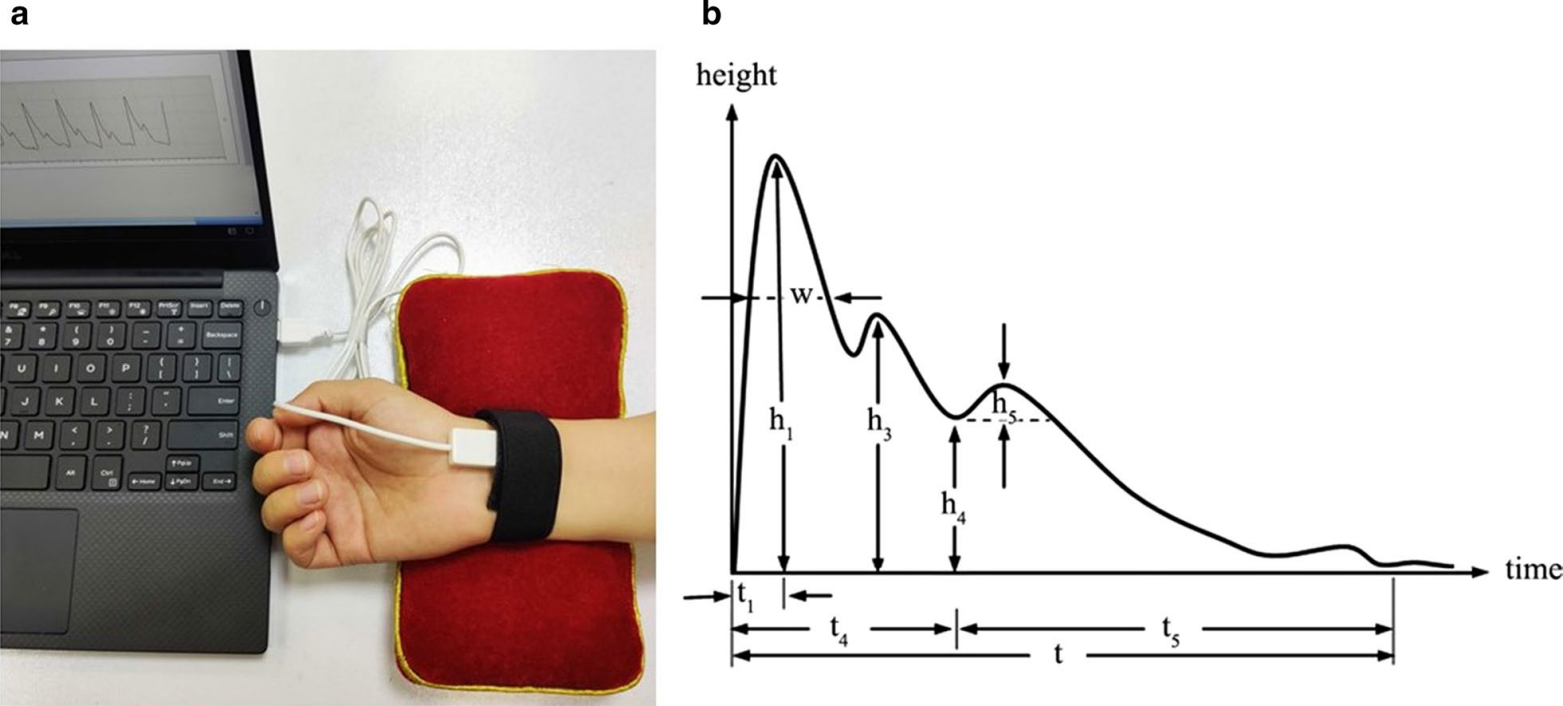
Figures of PDA-1 pulse diagnosis instrument and sphygmogram. **a** PDA-1 pulse diagnosis instrument and supporting equipment. **b** Sphygmogram of PDA-1 pulse diagnosis instrument

The four-diagnostic scale of TCM included 25 categories and 256 subitems. The symptoms and signs were classified as none, mild, and severe with 0, 1, and 2 points. We used Tongue and Face Diagnosis Analysis-1 instrument (TFDA-1) (software copyright registration No.: 2018SR033451) and Pulse Diagnosis Analysis-1 instrument (PDA-1) (Patent No.: ZL201620157027.6), which independent developed by the National Key Research and Development Program to collect the clinical tongue and pulse data. Besides, clinical Western medicine indexes mainly include 191 items such as blood routine, urine routine, liver and kidney function, tumor markers, electrocardiogram, and imaging examination. Considering fatigue had no specific indexes, the existing Western medicine indexes were auxiliary. For example, anemia fatigue patients were often associated with decreased hemoglobin, cancer fatigue patients were often associated with abnormal tumor markers. It was necessary to combine specific symptoms in specific diseases for comprehensive analysis and judgment. The tongue and face diagnosis equipment and the analysis interface were shown in Figs. 2 and 3. Figure 2a, b were the front and profile view of the instrument. The pulse diagnosis instrument and sphygmogram were shown in Fig. 4. Figure 4a was the PDA-1 pulse diagnosis instrument and supporting equipment. Figure 4b was the sphygmogram of PDA-1 pulse diagnosis instrument.

The color parameters of tongue image in Fig. 3 came from four color spaces: [37–39] RGB, HSI, Lab, and YCrCb. They were R (red), G (green) and B (blue), H (hue), S (saturation), I (brightness), L (lightness), a (red-green axis), b (yellow-blue axis), Y (brightness), Cr (the difference between the red part of the RGB input signal and the brightness value of the RGB signal), Cb (the difference between the blue part of RGB input signal and the brightness value of RGB signal), perAll (the ratio of the coating area to the total tongue area) and perPart (the ratio of the coating area to the non-coating tongue area).

The parameters of sphygmogram in Fig. 4b, h represented amplitude height, h_1_ was the main amplitude, h_3_ was the heavy wave front wave amplitude, h_4_ was the dicrotic notch amplitude, h_5_ was the gravity wave amplitude, t_1_ was the time value from the start point to the crest point of the main wave, t_4_ was the time value from the start point to the dicrotic notch, t_5_ was the time value from the dicrotic notch to the endpoint, t was one pulsating period, and w was divided into w_1_ and w_2_, w_1_ was 1/3 height of the main wave, w_2_ was 1/5 height of the main wave.

### Normalized data entry and extraction

Using Python3.7 to make data arrangement, and established the symptom and Western medicine index data sets, respectively. A total of 494 symptoms and indexes were collected, including 254 symptoms of TCM and 240 Western medicine indexes. The data sets were binarized. The positive TCM syndromes were recorded as "1", and negative TCM syndromes were recorded as "0". Negative qualitative data of Western medicine index were recorded as "0", positive data including weak positive (+) and strong positive (++ or +++) were recorded as "1", quantitative data in the normal range were recorded as "0", higher than or lower than normal range were recorded as "1".

### Screening of Symptoms and Indexes

According to the data characteristics in this study, the improved node contraction method was used to analyze the network nodes quantitatively. Node contraction was the integration of k nodes connected to the node with this node, replace the k + 1 nodes with a new node, and the edges that were previously associated with k + 1 nodes were now associated with the new node. After nodes with high importance were shrunk and fused, the whole network's connection would be closer, and the aggregation degree would increase. This method's basic idea was to shrink the nodes in the network one by one and then compare the network aggregation degree changes to rank the importance of nodes. The improved node contraction method [40] comprehensively considered the weight of edges in the weighted network. In this study, nodes represented symptoms or indexes, when two abnormal symptoms or indexes appeared in the same individual simultaneously, a connection was established between the two nodes. The weight of the edge represented the number of simultaneous occurrences of the two connected nodes. The larger the weight was, the closer the relationship between the two indexes. So here, we choose the sum of the edge weight as the point weight. In the weighted network, the corresponding concept of node degree was node strength. With the specific definition of node strength, extend the definition of network cohesion to weighted networks. Network cohesion refers to the reciprocal of the product of the number of nodes and the average shortest distance. Quantitatively describe the degree of network cohesion, weighted network cohesion [41] was defined as in formula (1).

1 $$\partial ({\text{WG}}) = \frac{1}{s \times l} = \frac{1}{{\mathop \sum \nolimits_{1}^{n} N_{i} \mathop \sum \nolimits_{{j \in N_{i} }} W_{ij} \times \frac{{\mathop \sum \nolimits_{i \ne j \in V} d_{ij} }}{n \times (n - 1)}}}$$

In the above formula, $s$ was the sum of the network's average node strength, which was the strength of each node divided by the number of neighboring nodes of the node. $l$ was the average shortest distance of the unweighted network corresponding to the weighted network after thresholding. *d*_*ij*_ was the shortest distance between node *V*_*i*_ and node *V*_*j*_ in the network. *W*_*ij*_ was the weight between node *V*_*i*_ and node *V*_*j*._ The node importance was expressed by IMC(*V*_*i*_), which was defined as in formula (2), ∂(WG ∗ V*i*) was the aggregation degree of the weighted network after contraction of node *V*_*i*_.

2 $${\text{IMC}}(V_{i} ) = 1 - \frac{\partial (WG)}{{\partial (WG \times Vi)}}$$

In this study, the improved node contraction method took the degree, betweenness, and edge weight of nodes, which was basically consistent with the purpose and requirement of each node's importance. In this paper, the sum of edge weight was used as the point weight to obtain an undirected weighted network. Triple was an improved form of adjacency list, and many network data were represented in the form of triples. A triple could be thought of as a three-column matrix, with each row in the format of [*V*_*i*_, *V*_*j*_, *W*_*ij*_], indicating the existence of an edge with a weight of W from node *V*_*i*_ to node *V*_*j*_. To better demonstrate the interaction between nodes, the symptom and index pairs were divided by the triad's maximum weight to obtain the normalized weight value.

### Construction and analysis of complex network

MATLAB (R2016a) software was used to process the binary data, and the core symptom and index data were selected according to the importance of node. Selected the top 10 data to construct the network and do analysis by Pajek (Pajek 64 5.08) software. The networks were output after editing each node's color and label and adjusting the node position manually.

### Statistical analysis

SPSS (Version 25.0) software was used for statistical analysis. Continuous data with normal distribution were presented as the mean and standard deviation, and those with abnormal distribution were presented as median and upper and lower quartiles. The categorical variables were expressed as counts and percentages. Analysis of Variance (ANOVA) was performed for data that normal distribution and homogeneity of variance among groups, Kruskal–Wallis H test was performed for non-normal distribution data. All the tests were two-tailed, and a *P* value < 0.05 was considered statistically significant.

### Quality control

In this study, researchers from Shanghai University of Traditional Chinese Medicine completed all scales and tongue and pulse collection. All researchers were medical professionals in TCM or integrated TCM and Western medicine. Moreover, all researchers have been trained in standard operating procedures to ensure consistency and accuracy in interpreting data collection results. Each study participant was interviewed by at least two professional researchers and supervised by at least two senior physicians to ensure data collection consistency and authenticity and reduce the measurement bias.

## Results

### Data set for fatigue group

There were 742 people in the healthy controls, 361 people in the sub-health fatigue group, and 1529 people in the disease fatigue group. The patients in the disease fatigue group were mainly hypertension, diabetes, hyperlipidemia, and fatty liver. The statistical analysis of baseline characteristics of the healthy controls, sub-health fatigue, and disease fatigue groups were shown in Table 1. "N" represented the number of categorical variables, "X ± SD" represented the mean and standard deviation of the continuous data Age and Body Mass Index (BMI).

**Table 1:** Statistical analysis of baseline characteristics

Group	N	Male	Female	Age	BMI (kg/m^2^)
		N (%)	N (%)	(X ± SD, year)	
Healthy controls	742	553 (74.5)	189 (25.5)	32.52 ± 10.16	22.71 ± 3.08
Sub-health fatigue group	361	215 (59.6)	146 (40.4)	34.64 ± 9.45**	22.74 ± 3.46
Disease fatigue group					
Hypertension	311	228 (73)	83 (27)	48.56 ± 13.94**^##^	25.51 ± 3.41**^##^
Diabetes	157	127 (81)	30 (19)	54.04 ± 12.79**^##^	25.95 ± 3.67**^##^
Hyperlipemia	518	373 (72)	145 (28)	45.87 ± 12.69**^##^	24.97 ± 3.27**^##^
Fatty liver	442	334 (76)	108 (24)	45.10 ± 13.20**^##^	26.57 ± 3.06**^##^

*versus healthy controls, *P* < 0.05

**versus healthy controls, *P* < 0.01

^#^versus sub-health fatigue group, *P* < 0.05

^##^versus sub-health fatigue group, *P* < 0.01

From the result, we could see that there were many more males than females in the three groups, actually the total number of people who participated in medical examinations, males were higher than females, the reason might be that there were more males than females in routine physical examinations in the area where the hospital was located, and males might pay more attention to routine physical examinations. Age and BMI were statistically significant in the sub-health fatigue group and disease fatigue group subjects compared with healthy controls (*P* < 0.01), and age was statistically significant in the disease fatigue group compared with the sub-health fatigue group (*P* < 0.01).

### Construct and analyze of symptom network of the sub-health fatigue group

**Fig.5:**
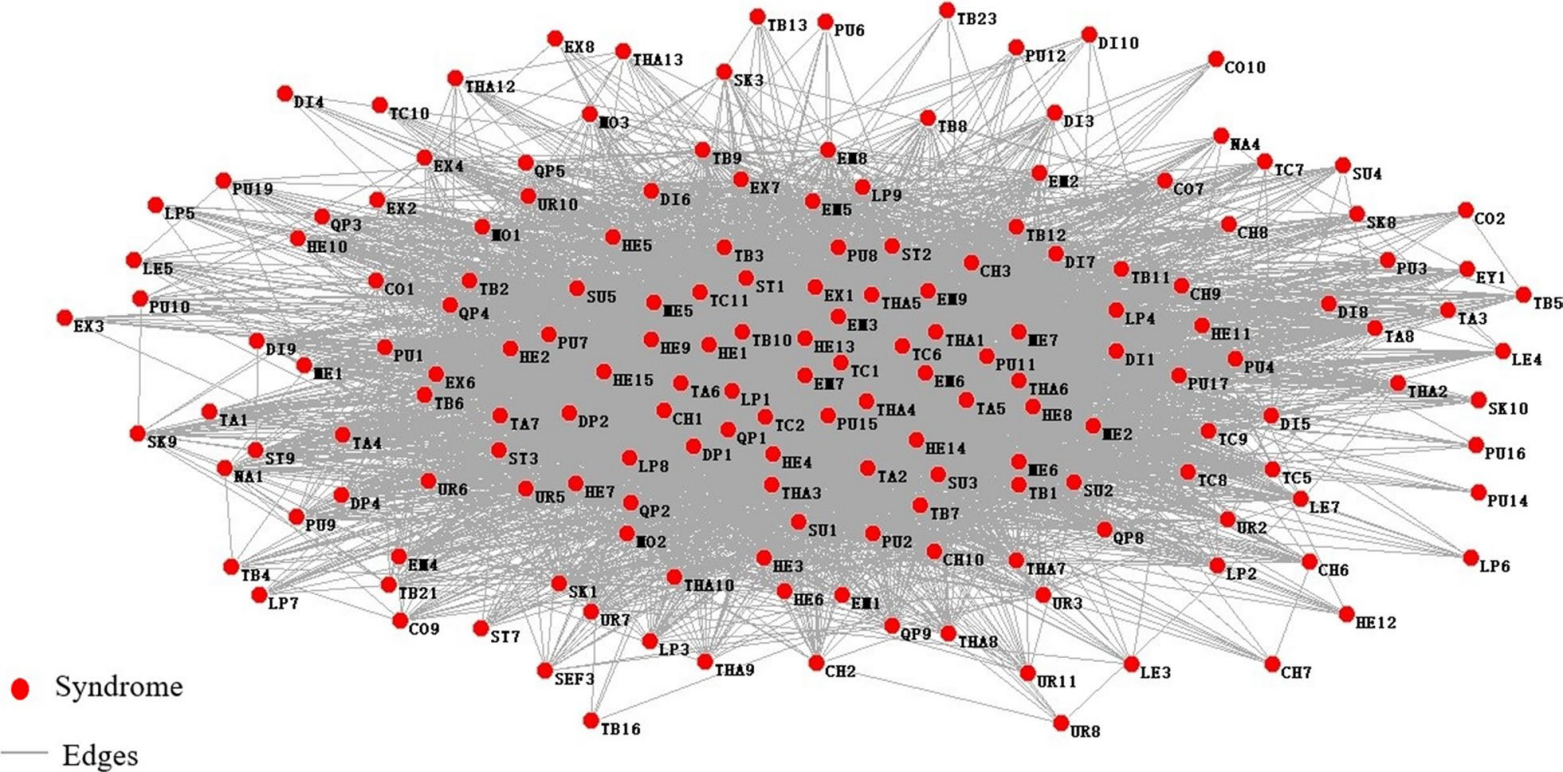
Symptom network of the sub-health fatigue group

Using MATLAB for data processing of sub-health fatigue group with the whole symptoms. Binary data of TCM symptoms were converted into ". NET" format and then using Pajek software to draw the networks, and the symptom network was shown in Fig. 5.

**Fig.6:**
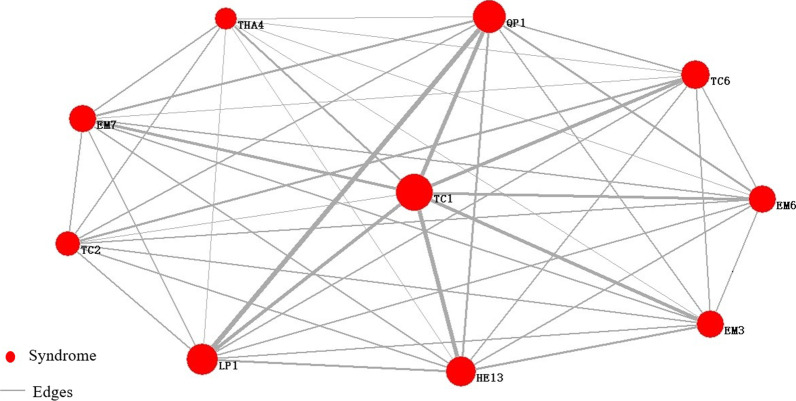
Network of core symptom of the sub-health fatigue group. TC1: white tongue coating, LP1: headache, TC2: yellow tongue coating, QP1: sour, EM7: dreaminess, EM3: irritability, THA4: chest distress, HE13: xerophthalmia, TC6: thick coating, EM6: insomnia

As the total network had many nodes and complex network relationships, its core nodes' relationships could not be well described. Therefore, it was necessary to select core nodes according to the importance of nodes IMC(V_i_), and then used Pajek software to draw the core symptoms network. The network was shown in Fig. 6. In the network, the node's size represented the strength, the thickness of the edge represented the weight between nodes, and the core symptoms were shown in Table 2.

**Table 2:** Core symptoms of sub-health fatigue group

Index	Symptom	IMC (V_i_)
TC1	White tongue coating	0.999
LP1	Headache	0.997
TC2	Yellow tongue coating	0.996
QP1	Sour	0.996
EM7	Dreaminess	0.994
EM3	Irritability	0.994
THA4	Chest distress	0.992
HE13	Xerophthalmia	0.991
TC6	Thick coating	0.991
EM6	Insomnia	0.989

To analyze the network, took the symptom associated pairs whose normalized weight was greater than 0.5, and the associated result was shown in Table 3.

**Table 3:** Associated analysis of core symptoms of sub-health fatigue group

Symptom		Weight
LP1	QP1	1.000
TC1	QP1	0.905
	HE13	0.833
	LP1	0.810
	TC6	0.750
	EM3	0.679
	EM6	0.643
	EM7	0.631
TC2	TC6	0.512
QP1	HE13	0.464

### Construct and analyze of symptom and index networks of the disease fatigue group

**Fig.7:**
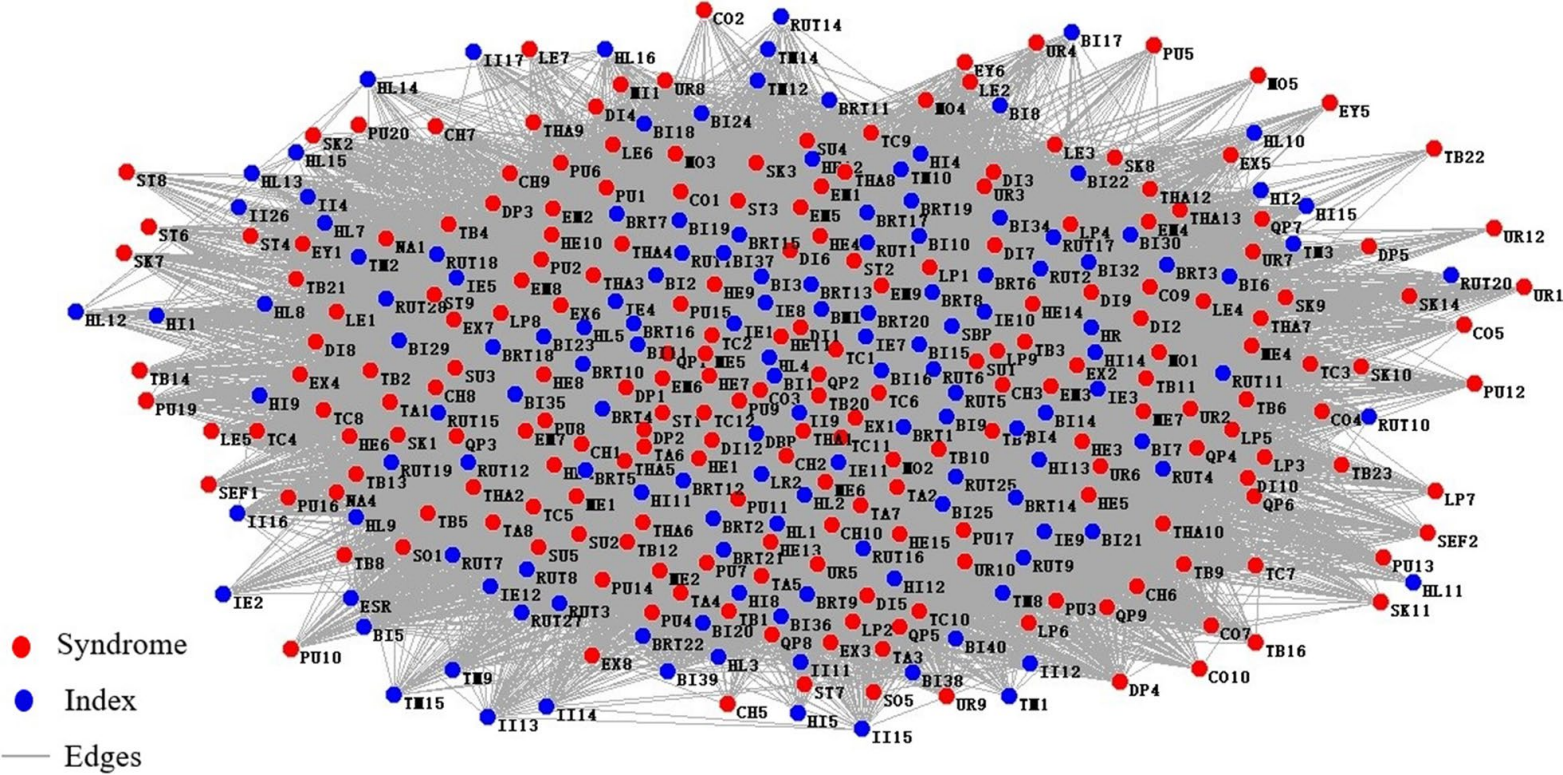
Symptom and index network of the disease fatigue group

**Fig.8:**
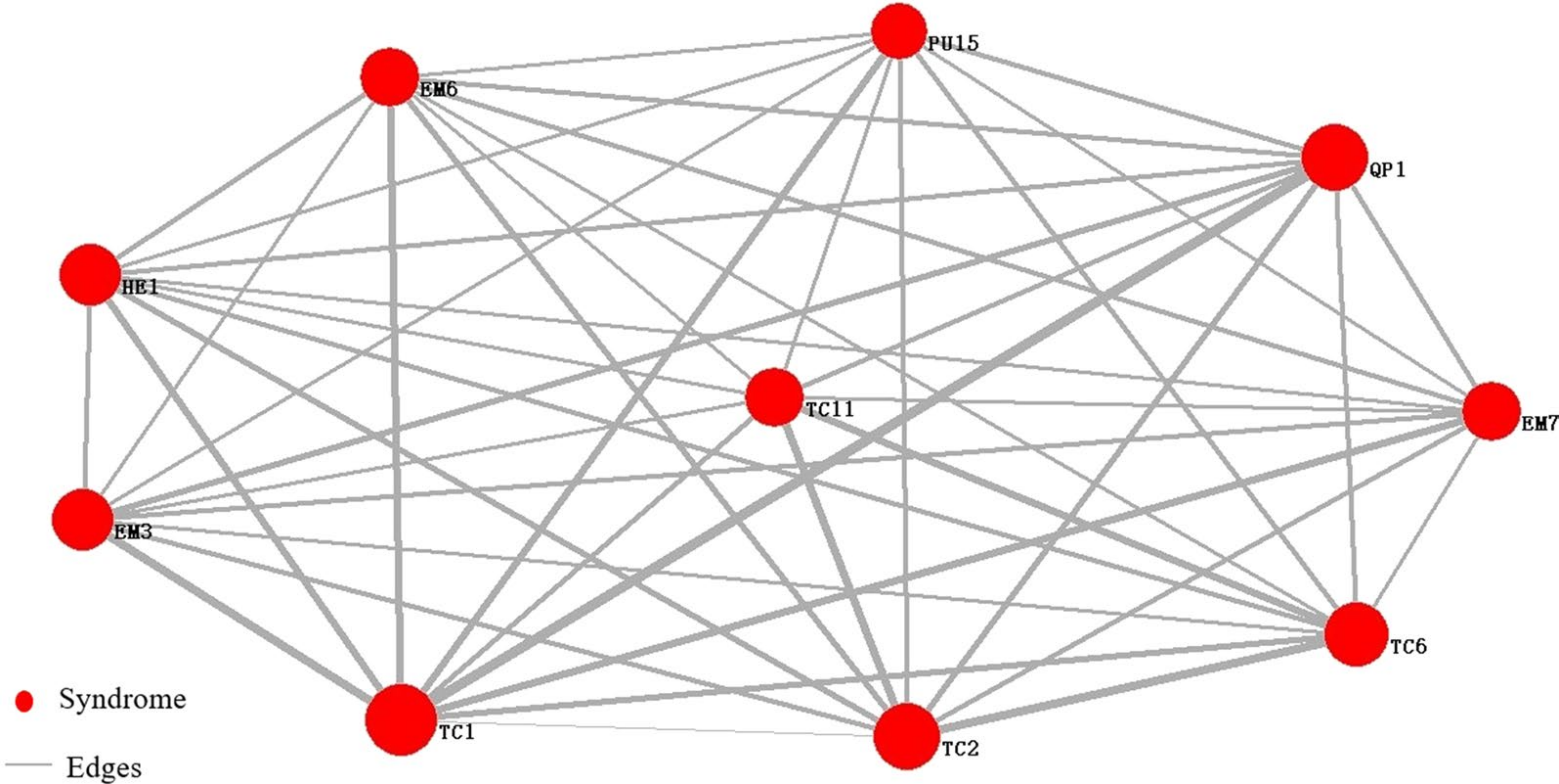
Network of core symptom of the disease fatigue group. TC1: white tongue coating, HE1: dizziness, TC2: yellow tongue coating, QP1: sour, EM7: dreaminess, TC6: thick coating, PU15: string-like pulse, TC11: greasy coating, EM6: insomnia, EM3: irritability

**Fig.9:**
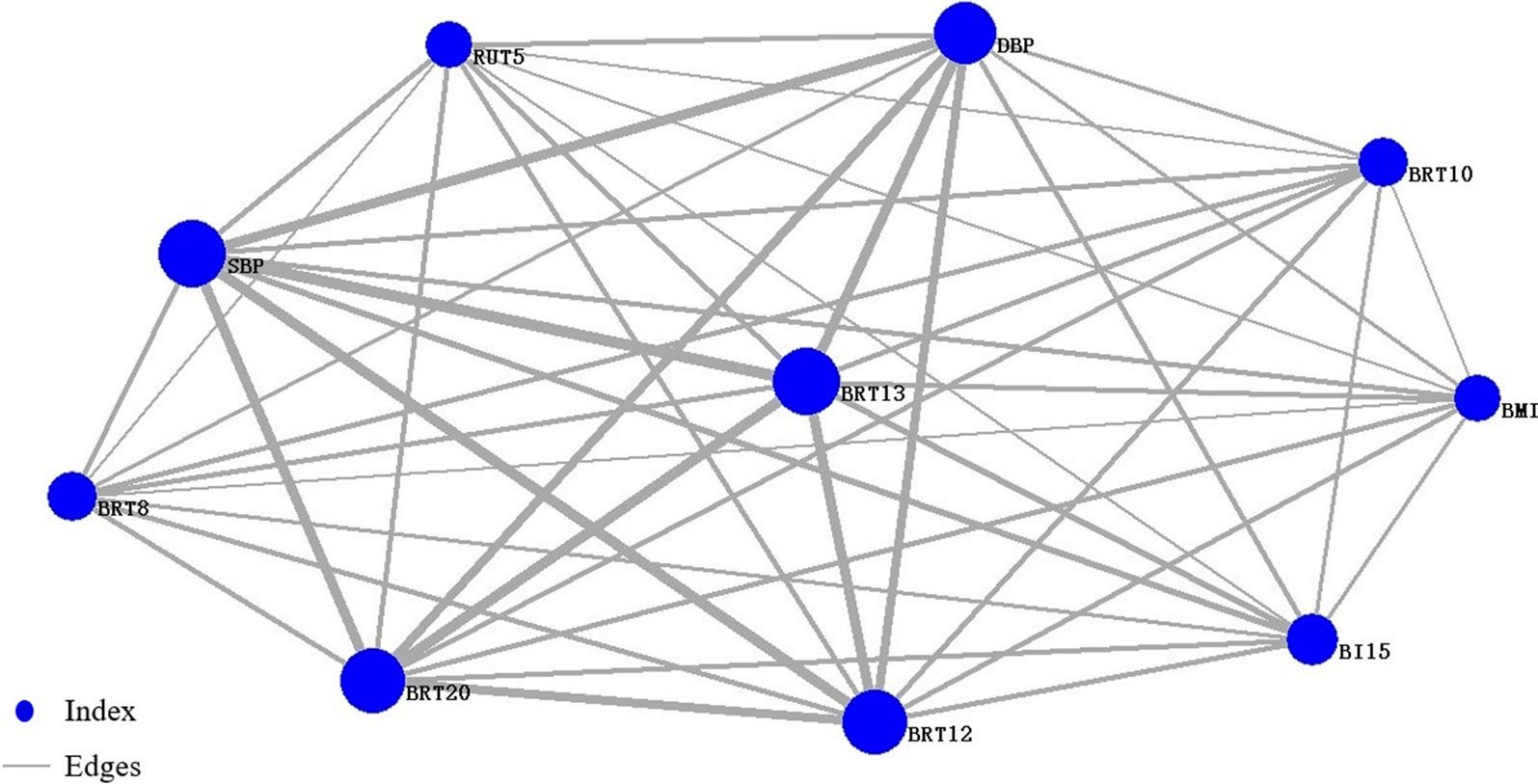
Network of core index of the group of disease fatigue. BRT13: basophil, BRT20: platelet distribution width, SBP: systolic blood pressure, BRT12: percentage of monocyte, DBP: diastolic blood pressure, RUT5: PH of urine, BRT8: hemoglobin, BRT10: hematocrit, BI15: uric acid, BMI: body mass index

Using the same method to draw a network of the whole symptom and index of disease fatigue population, as shown in Fig. 7. To Select core symptoms and indexes and draw networks of disease fatigue group. Core symptom and index networks were shown in Figs. 8 and 9. The core symptoms and indexes and node importance rank were shown in Tables 4 and 5.

**Table 4:** Core symptoms and node importance rank of the disease fatigue group

Index	Symptom	IMC (V_i_)
TC1	White tongue coating	1.000
HE1	Dizziness	1.000
TC2	Yellow tongue coating	1.000
QP1	Sour	1.000
EM7	Dreaminess	1.000
TC6	Thick coating	1.000
PU15	Wiry pulse	1.000
TC11	Greasy coating	1.000
EM6	Insomnia	1.000
EM3	Irritability	0.999

**Table 5:** Core indexes and node importance rank of the disease fatigue group

Index	Index	IMC (V_i_)
BRT13	Basophil	1.000
BRT20	Platelet distribution width	1.000
SBP	Systolic blood pressure	1.000
BRT12	Percentage of monocyte	1.000
DBP	Diastolic blood pressure	1.000
RUT5	PH of urine	1.000
BRT8	Hemoglobin	1.000
BRT10	Hematocrit	1.000
BI15	Uric acid	1.000
BMI	Body mass index	1.000

**Fig.10:**
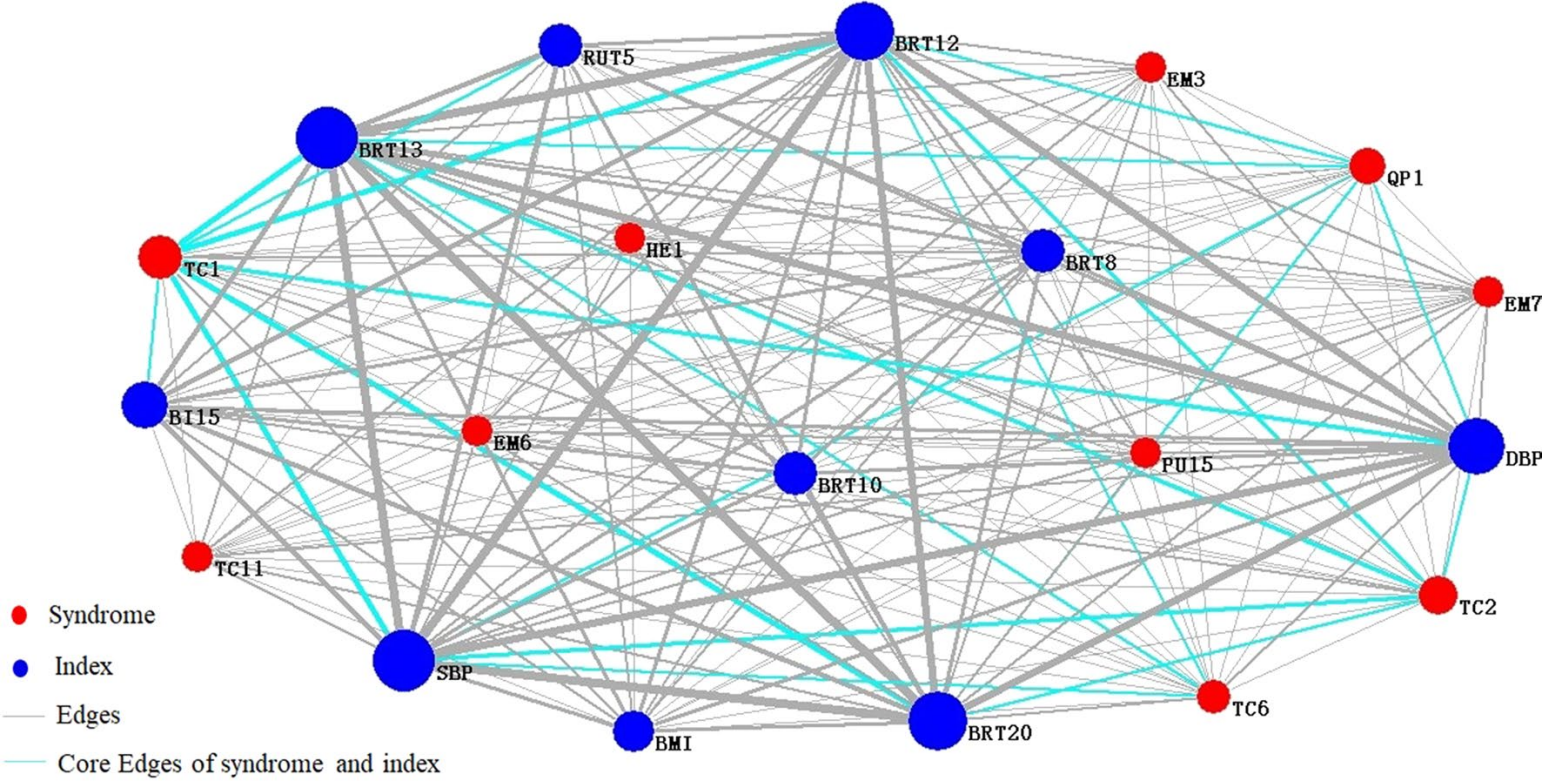
Network of core symptom-indicator of the group of disease fatigue. TC1: white tongue coating, HE1: dizziness, TC2: yellow tongue coating, QP1: sour, EM7: dreaminess, TC6: thick coating, PU15: wiry pulse, TC11: greasy coating, EM6: insomnia, EM3: irritability, BRT13: basophil, BRT20: platelet distribution width, SBP: systolic blood pressure, BRT12: percentage of monocyte, DBP: diastolic blood pressure, RUT5: PH of urine, BRT8: hemoglobin, BRT10: hematocrit, BI15: uric acid, BMI: body mass index

Relationships between symptoms and indexes were very complicated in the actual clinical diagnosis of disease. The diagnosis could not rely on symptoms or indexes solely. It was necessary to combine them to analyze together. Its core symptom-index interaction edges were shown as cyan lines. The core symptom-index network was shown in Fig. 10.

To analyze the network, took the top 10 pairs of core symptom-symptom pairs and index-index pairs, respectively, and the associated analysis results were shown in Tables 6 and 7.

**Table 6:** Associated analysis of core symptom-symptom pairs of the disease fatigue group

Symptom		Weight
TC1	QP1	0.187
TC2	TC6	0.167
TC1	EM3	0.153
TC2	TC11	0.149
TC1	HE1	0.142
TC1	EM7	0.137
PU15	TC1	0.134
TC1	EM6	0.132
TC6	TC11	0.129
TC2	QP1	0.123

**Table 7:** Associated analysis of core index-index pairs of the disease fatigue group

Index		Weight
BRT13	SBP	1.000
BRT13	BRT20	0.921
BRT13	BRT12	0.913
BRT20	SBP	0.908
SBP	BRT12	0.902
DBP	SBP	0.873
BRT13	DBP	0.868
BRT20	BRT12	0.824
DBP	BRT12	0.782
BRT20	DBP	0.782

To select the core symptom-index associated results, the top 10 symptom-index pairs were shown in Table 8.

**Table 8:** Associated analysis of core symptom-index of the disease fatigue group

Symptom	Index	Weight
TC1	SBP	0.546
	BRT13	0.545
	BRT20	0.494
	BRT12	0.491
	DBP	0.474
TC2	BRT13	0.376
	SBP	0.372
	BRT12	0.342
	BRT20	0.338
	DBP	0.326

In conclusion, the research results showed that white coating, yellow coating, sour, dreaminess, irritability, thick coating, and insomnia were the common symptoms of the two groups of fatigue population. The main difference was that the node importance of the same symptom was different in different fatigue population networks, indicating that the diagnostic contribution rate of the same symptom to different fatigue population was different. Headache, chest distress, and xerophthalmia were more significant in sub-health fatigue group, while dizziness, wiry pulse, and greasy coating were more significant in disease fatigue group. The most common abnormal indexes in the disease fatigue group were basophil, platelet distribution width, systolic blood pressure, percentage of monocyte, diastolic blood pressure, PH of urine, hemoglobin, hematocrit, uric acid, and body mass indicator. The symptom-index associated analysis showed that systolic blood pressure, basophil, platelet distribution width, percentage of monocyte, and diastolic blood pressure were closely related to white coating, and it was also related to yellow coating to some extent.

### Canonical correlation analysis of tongue and pulse parameters

Using SPSS (Version 25.0) software to detect outliers or extreme values. Individuals with outliers or extreme values in tongue or pulse data of the three groups were excluded, although this reduced the sample size to some extent, it ensured the accuracy of the results. Finally, 551 people were included in the healthy controls, 252 in the sub-health fatigue group, and 1160 in the disease fatigue group.

**Fig.11:**
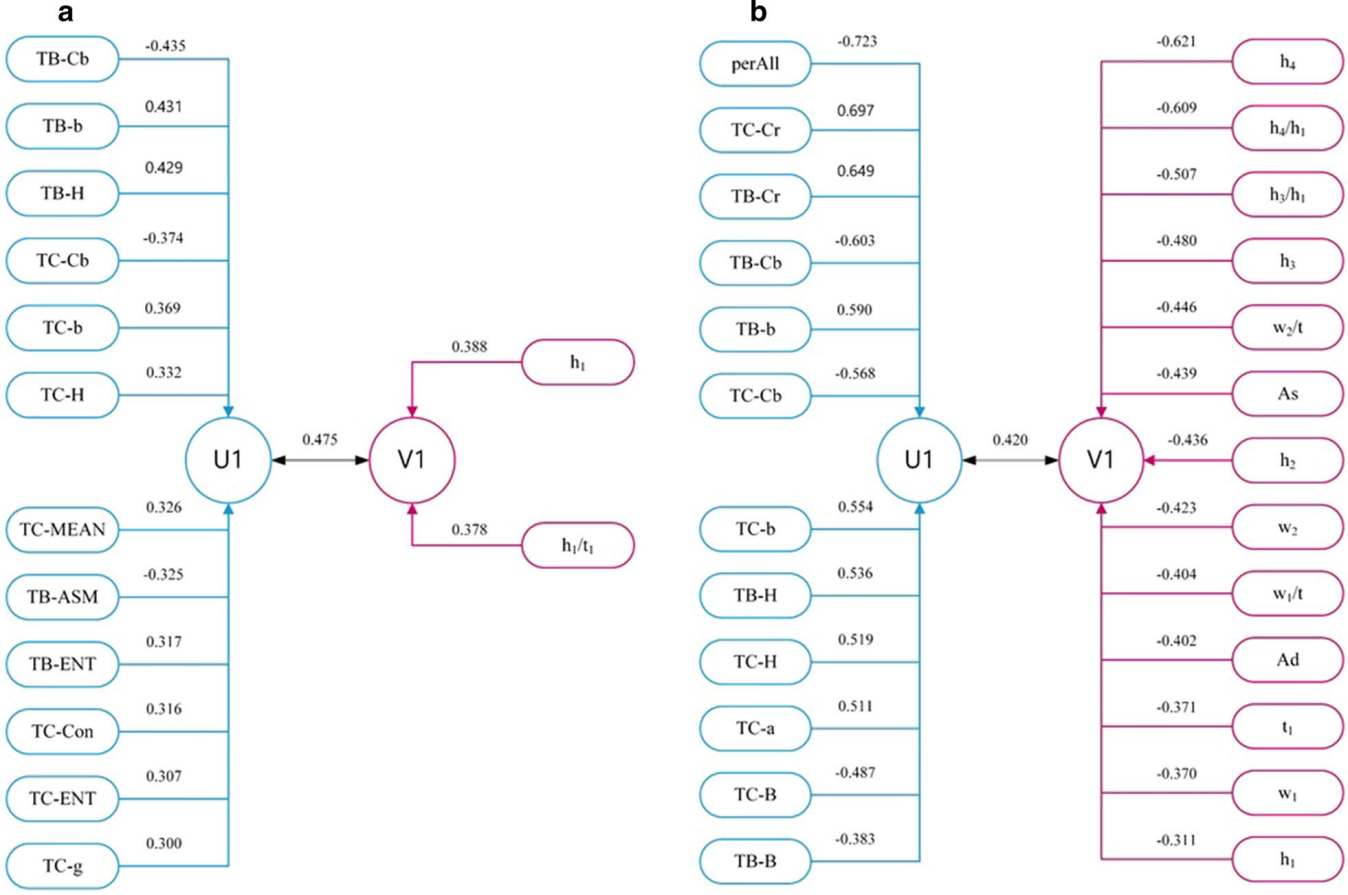
Structure diagram of canonical correlation analysis of tongue and pulse parameters. The left indexes of **a** and **b** are the parameters of tongue, the right indexes are the parameters of pulse. The prefix TB represents the tongue body index, the prefix TC represents the tongue coating index. U1 is the representative comprehensive variable extracted from the tongue parameters, V1 is the representative comprehensive variable extracted from the pulse parameters. **a** Healthy controls. **b** Disease fatigue group

Canonical Correlation Analysis verified the overall correlation between one set of variables and another. The results showed a certain correlation between the tongue and pulse data in healthy controls and the disease fatigue group. The correlation coefficient of tongue and pulse data in healthy controls was 0.475 (*P* < 0.05), tongue characteristic parameters were mainly affected by TB-Cb, TB-b, TB-H, and TC-Cb, the canonical correlation coefficients were − 0.435, 0.431, 0.429, and − 0.374, respectively, *P* < 0.05. Pulse characteristic parameters were mainly affected by h_1_ and h_1_/t_1_, the canonical correlation coefficients were 0.388 and 0.378, respectively, *P* < 0.05, as shown in Fig. 11a. The correlation coefficient of tongue and pulse data in disease fatigue group was 0.420 (*P* < 0.05), tongue characteristic parameters were mainly affected by perAll, TC-Cr, TB-Cr, TB-Cb, TB-b, and the canonical correlation coefficients were − 0.723, 0.697, 0.649, − 0.603 and 0.590, respectively, *P* < 0.05. Pulse characteristic parameters were mainly affected by h_4_, h_4_/h_1_, h_3_/h_1_, h_3_ and w_2_/t, the canonical correlation coefficients were − 0.621, − 0.609, − 0.507, − 0.480 and − 0.446, respectively, *P* < 0.05, as shown in Fig. 11b. There was no statistically significant correlation between the tongue and pulse data in sub-health fatigue group.

## Discussion

Fatigue is an important early warning signal of abnormal health status. It should be treated in time to prevent it from further developing into more serious diseases. At present, there are two main reasons for the difficulties in fatigue research. Firstly, the mechanism of fatigue is complex, and there is still a lack of diagnostic criteria for fatigue. Secondly, there is a lack of effective fatigue evaluation models [42]. Modern tongue and pulse diagnosis can provide good data support, coupled with the assistance of artificial intelligence and technical machine learning technology, providing new methods and ideas for accurate diagnosis of fatigue.

In this study, complex network technology was used to screen out the main symptoms and indexes of fatigue patients and the interaction between symptoms and indexes. Studying the interrelationship between fatigue-related symptoms contributed to further determine the direction of diagnosis of fatigue-related diseases. Headache, chest distress, and xerophthalmia were more significant in sub-health fatigue population. Headache and chest distress were generally manifested as qi stagnation syndrome, and xerophthalmia was the common clinical manifestation of jinye deficiency syndrome. Wiry pulse, greasy coating, and dizziness were more significant in disease fatigue group. The clinical significance of wiry pulse is mainly about liver and gallbladder disease, pain, phlegm and retained fluid, consumptive disease, and stomach gas decline. The clinical significance of greasy coating was phlegm-damp, phlegm and retained fluid, and dyspepsia. These two symptoms were consistent with common pathological manifestations of the disease. Furthermore, dizziness was the concomitant symptom of hypertension, hypoglycemia, anemia, and cancer. Basophil, platelet distribution width, percentage of monocyte, hemoglobin, hematocrit were blood routine item, the value of PH of urine and uric acid are routine items of urine examination, abnormal of these two indexes mostly indicate the abnormal renal function, and BMI mostly reflects human metabolism, so it can be seen that abnormal blood routine, renal function, blood pressure, and basic metabolism are more common in patients with fatigue.

Associated analysis of symptoms and indexes can better explore the nature of diseases [43, 44]. In this study, the symptom-index associated analysis showed that systolic blood pressure, basophil, platelet distribution width, percentage of monocyte, and diastolic blood pressure were closely related to white coating and yellow coating to some extent. The clinical significance of white coating in disease state was mainly surface syndrome, cold syndrome, and dampness syndrome. Thus, the fatigue population were mostly seen in surface syndrome, cold syndrome, and dampness syndrome. This analysis was helpful for better understanding the core symptoms, the interaction between symptoms, and the distribution of syndromes of fatigue population to provide a theoretical basis for the rapid and accurate diagnosis.

Fatigue as a comprehensive performance of the whole body, it was necessary to analyze the relationship of indexes. The canonical correlation analysis was used for the combined analysis of tongue and pulse data. Canonical correlation analysis of tongue and pulse data showed that the healthy controls' correlation was stronger than that in the disease fatigue group. In contrast, the correlation coefficient between canonical variables and all tongue and pulse variables in the disease fatigue group was greater than that in the healthy controls. The reason might be that there are many kinds of diseases in disease fatigue group while the healthy controls were relatively single. The tongue and pulse in the healthy controls tended to be more stable, and its characteristics were relatively stable. For example, the healthy controls' tongue was generally reddish tongue, thin white tongue coating, and the pulse was usually normal pulse. The tongue and pulse in the disease fatigue group might be diversified due to different diseases. Patients' tongue could present as purple-red tongue, bluish-purple tongue, yellow greasy tongue coating, white greasy tongue coating, etc. Their pulse could be different forms of wiry pulse, tight pulse, slippery pulse, uneven pulse, etc. The tongue and pulse abnormalities of disease fatigue population destroyed a certain stable correlation of the health state and tended to a certain partial correlation.

This study still has some limitations. Firstly, there are many kinds of diseases in this study, which did not conducive to interpreting the results. In the future, specific diseases will can be refined to analyze the relationship of symptom-index and the tongue-pulse data. Secondly, complexion spectral data can be added based on tongue and pulse data. Integrating more objective indexes that can objectively evaluate fatigue will be more productive to analyze its phenomenon and mechanism. Besides, this study still lacks treatment guidance and intervention for fatigue population, which will be improved in the future.

## Conclusion

In summary, this study constructed the fatigue-related symptom networks and symptom-index networks, analyzed the data relationship of tongue and pulse in fatigue population, and revealed the distribution rule of symptom and index, the tongue and pulse data in different fatigue population. It provided an objective basis for establishing the data evaluation of fatigue state, and we are looking forward to establishing a fatigue evaluation method based on objective data of tongue and pulse in the future.

## Data Availability

The datasets generated and analyzed during the current study are not publicly available due to the confidentiality of the data, which is an important component of the National Key Technology R&D Program of the 13th Five-Year Plan (No. 2017YFC1703301) in China, but are available from the corresponding author on reasonable request.

## Abbreviations

TCM: Traditional Chinese Medicine
PDA-1: Pulse Diagnosis Analysis-1
TFDA-1: Tongue Face Diagnosis Analysis-1
ANOVA: Analysis of Variance
BMI: Body Mass Index
TB: Tongue Body
TC: Tongue Coating

## References

[1] Chaudhuri A, Behan PO (2004). Fatigue in neurological disorders. Lancet.

[2] Kim S, Jang HJ, Myung W, Kim K, Cha S, Lee H (2019). Heritability estimates of individual psychological distress symptoms from genetic variation. J Affect Disord.

[3] Kluger BM, Herlofson K, Chou KL, Lou JS, Goetz CG, Lang AE (2016). Parkinson's disease-related fatigue: A case definition and recommendations for clinical research. Mov Disord.

[4] Chung KF, Yu YM, Yeung WF (2015). Correlates of residual fatigue in patients with major depressive disorder: the role of psychotropic medication. J Affect Disord.

[5] Skorvanek M, Gdovinova Z, Rosenberger J, Saeedian RG, Nagyova I, Groothoff JW (2015). The associations between fatigue, apathy, and depression in Parkinson's disease. Acta Neurol Scand.

[6] Lu Y, Qu HQ, Chen FY, Li XT, Cai L, Chen S (2019). Effect of Baduanjin qigong exercise on cancer-related fatigue in patients with colorectal cancer undergoing chemotherapy: a randomized controlled trial. Oncol Res Treat.

[7] Grad FP (2002). The preamble of the constitution of the World Health Organization. Bull World Health Organ.

[8] Xue Y, Liu G, Feng Y, Xu M, Jiang L, Lin Y (2020). Mediating effect of health consciousness in the relationship of lifestyle and suboptimal health status: a cross-sectional study involving Chinese urban residents. BMJ Open.

[9] Wang X, Liu J, Wu C, Liu J, Li Q, Chen Y (2020). Artificial intelligence in tongue diagnosis: Using deep convolutional neural network for recognizing unhealthy tongue with tooth-mark. Comput Struct Biotechnol J.

[10] Li X, Zhang Y, Cui Q, Yi X, Zhang Y (2019). Tooth-marked tongue recognition using multiple instance learning and CNN features. IEEE Trans Cybern.

[11] Qin B, Liang L, Wu J, Quan Q, Wang Z, Li D (2020). Automatic identification of down syndrome using facial images with deep convolutional neural network. Diagnostics (Basel).

[12] Pan Z, Shen Z, Zhu H, Bao Y, Liang S, Wang S (2020). Clinical application of an automatic facial recognition system based on deep learning for diagnosis of turner syndrome. Endocrine.

[13] Tang AC, Chung JW, Wong TK (2012). Digitalizing traditional Chinese medicine pulse diagnosis with artificial neural network. Telemed J E Health.

[14] Hu MC, Cheng MH, Lan KC (2016). Color correction parameter estimation on the smartphone and its application to automatic tongue diagnosis. J Med Syst.

[15] Zhang B, Wang X, You J, Zhang D (2013). Tongue color analysis for medical application. Evid Based Complement Alternat Med.

[16] Hu XJ, Zhang L, Xu JT, Liu BC, Wang JY, Hong YL (2018). Pulse wave cycle features analysis of different blood pressure grades in the elderly. Evid Based Complement Alternat Med.

[17] Luo ZY, Cui J, Hu XJ, Tu LP, Liu HD, Jiao W (2018). A study of machine-learning classifiers for hypertension based on radial pulse wave. Biomed Res Int.

[18] Wang X, Zhang B, Yang Z, Wang H, Zhang D (2013). Statistical analysis of tongue images for feature extraction and diagnostics. IEEE Trans Image Process.

[19] Kamarudin ND, Ooi CY, Kawanabe T, Odaguchi H, Kobayashi F (2017). A fast SVM-based tongue's colour classification aided by k-means clustering identifiers and colour attributes as computer-assisted tool for tongue diagnosis. J Healthc Eng.

[20] Zhang JF, Xu JT, Hu XJ, Chen QG, Tu L, Huang JB (2017). Diagnostic method of diabetes based on support vector machine and tongue images. Biomed Res Int.

[21] Ding T, Feng L, Rong L, Xi LD. Tongue inspection on fatigue. In: The 10th Annual conference of rehabilitation Committee of Traditional Chinese Medicine of China Disabled Persons' Rehabilitation Association; 2015. p. 4.

[22] Li WL, Yi ZX, Min P (2019). Objective analysis of complexion and tongue color in patients with chronic fatigue syndrome. Shandong Med J.

[23] Xu JT, Bao YM, Gong BM (2008). Experimental study on evaluation of sphygmogram of chronic motion fatigue. Shanghai J Tradit Chin Med.

[24] Kung YY, Kuo TBJ, Lai CT, Shen YC, Su YC, Yang CCH (2020). Disclosure of suboptimal health status through traditional Chinese medicine-based body constitution and pulse patterns. Complement Ther Med.

[25] Shi HZ, Fan QC, Gao JY, Liu JL, Bai GE, Mi T (2017). Evaluation of the health status of six volunteers from the Mars 500 project using pulse analysis. Chin J Integr Med.

[26] Li S (2011). Network target: a starting point for traditional Chinese medicine network pharmacology. Zhongguo Zhong Yao Za Zhi.

[27] Liu ZH, Sun XB (2012). Network pharmacology: new opportunity for the modernization of traditional Chinese medicine. Yao Xue Xue Bao.

[28] Wang ZF, Hu YQ, Wu QG, Zhang R (2019). Virtual screening of potential anti-fatigue mechanism of Polygonati Rhizoma based on network pharmacology. Comb Chem High Throughput Screen.

[29] Liu H, Zeng L, Yang K, Zhang G (2016). A network pharmacology approach to explore the pharmacological mechanism of Xiaoyao powder on anovulatory infertility. Evid Based Complement Alternat Med.

[30] Wu L, Gao X, Cheng Y, Wang Y, Zhang B, Fan X (2011). Symptom-based traditional Chinese medicine slices relationship network and its network pharmacology study. Zhongguo Zhong Yao Za Zhi.

[31] Zhang R, Zhu X, Bai H, Ning K (2019). Network pharmacology databases for traditional Chinese medicine: review and assessment. Front Pharmacol.

[32] Peckham AD, Jones P, Snorrason I, Wessman I, Beard C, Björgvinsson T (2020). Age-related differences in borderline personality disorder symptom networks in a transdiagnostic sample. J Affect Disord.

[33] Song J, Liu X, Deng Q, Dai W, Gao Y, Chen L (2015). A network-based approach to investigate the pattern of syndrome in depression. Evid Based Complement Alternat Med.

[34] Shi Q, Zhao H, Chen J, Ma X, Yang Y, Zheng C (2012). Study on TCM syndrome identification modes of coronary heart disease based on data mining. Evid Based Complement Alternat Med.

[35] Chen J, Lu P, Zuo X, Shi Q, Zhao H, Luo L (2012). Clinical data mining of phenotypic network in angina pectoris of coronary heart disease. Evid Based Complement Alternat Med.

[36] Henry TR, Marshall SA, Avis NE, Levine BJ, Ip EH (2018). Concordance networks and application to clustering cancer symptomology. PLoS ONE.

[37] Fernandez-Rodriguez J, Moser F, Song M, Voigt CA (2017). Engineering RGB color vision into *Escherichia coli*. Nat Chem Biol.

[38] Schiller F, Valsecchi M, Gegenfurtner KR (2018). An evaluation of different measures of color saturation. Vision Res.

[39] Sun X, Young J, Liu JH, Bachmeier L, Somers RM, Chen KJ (2016). Prediction of pork color attributes using computer vision system. Meat Sci.

[40] Zhu T, Zhang SP, Guo RX, Chang GC (2009). Improved evaluation method for node importance based on node contraction in weighted complex networks. Syst Eng Electron.

[41] Tan YJ, Wu J, Deng HZ (2006). Evaluation method for node importance based on node contraction in complex networks. SystEng Theory Pract.

[42] Enoka RM, Duchateau J (2016). Translating fatigue to human performance. Med Sci Sports Exerc.

[43] Yuan YH (2003). Relationship between tongue image and liver function in virus hepatitis patients—a report of 200 cases. Jiangsu J Tradit Chin Med.

[44] Lin RY, Yu HY, Qin JY, Li YY, Wang YH, Yang YZ (2015). Association between tongue coating thickness and clinical characteristics among idiopathic membranous nephropathy patients. J Ethnopharmacol.

